# Supplementary material for: The Ontology of Biological Attributes (OBA) - Computational Traits for the Life Sciences
Source: bioRxiv. 2023 Jan 27:2023.01.26.525742. Preprint. [Version 1] doi: 10.1101/2023.01.26.525742 (PMC9900877; doi:10.1101/2023.01.26.525742)
Supplement: Supplement 1 [file NIHPP2023.01.26.525742v1-supplement-1.pdf]

bioRxiv preprint doi: https://doi.org/10.1101/2023.01.26.525742; this version posted January 27, 2023. The copyright holder for this preprint (which was not certified by peer review) is the author/funder, who has granted bioRxiv a license to display the preprint in perpetuity. It is made available under aCC-BY 4.0 International license.

Supplementary Materials

S1: All DOS-DP patterns used by OBA

**Pattern name**	**Description**	**Example**
attribute_location	This pattern describes an attribute of a specified entity that can be observed, measured or quantified in a specific location.	OBA:0000091 lysosomal lumen pH
chemical_role_attribute	This pattern describes an attribute of a chemical entity, where the chemical entity is a category of chemicals with a specific role.	OBA:0000079 hormone levels
chemical_role_attribute_location	This pattern describes an attribute of a chemical entity in a specified location, where the chemical entity is a category of chemicals with a specific role.	OBA:VT0003369 blood estrogen amount
disease age of onset	Age at which disease manifestations first appear.	OBA:2001000 age of onset of Alzheimer disease
entity_attribute	This pattern describes an attribute of a specific entity that can be observed, measured or quantified.	OBA:0002360 trochanter size
entity_attribute_location	This pattern describes an attribute of a specific entity that can be observed, measured or quantified in a specified location.	OBA:2020005 lysine in blood amount
entity_attribute_part_of	This pattern describes very general grouping traits. This should be used only in conjunction with morphology and quality attributes.	OBA:1000013 cytoskeleton morphology
process_attribute_location	This pattern describes an attribute of a biological process in some location.	OBA:2050069 serum lipase activity amount

bioRxiv preprint doi: https://doi.org/10.1101/2023.01.26.525742; this version posted January 27, 2023. The copyright holder for this preprint (which was not certified by peer review) is the author/funder, who has granted bioRxiv a license to display the preprint in perpetuity. It is made available under aCC-BY 4.0 International license.

response_to_chemical_stimulus_trait	This pattern describes a trait that affects the biological response to a chemical.	OBA:2040012 trait in response to morphine
response_to_chemical_with_role_stimulus	This pattern describes a trait that affects the biological response to a chemical, where the chemical is a category of chemicals with a specified role.	OBA:2040009 trait in response to endocrine disruptor

S2: Supplementary query (SPARQL): Aggregate all data related to the morphology of the a part of the cardiovascular system

Note: This query works when *part_of (BFO:0000050)* and *subclass_of (rdfs:subclassOf)* edges are materialised, like in Ubergraph (https://ubergraph.apps.renci.org/sparql). Otherwise, wildcard operators (*) need to be added to the properties.

```
PREFIX subclass_of: <http://www.w3.org/2000/01/rdf-schema#subClassOf>
PREFIX label: <http://www.w3.org/2000/01/rdf-schema#label>
PREFIX characteristic_of: <http://purl.obolibrary.org/obo/RO_0000052>
PREFIX part_of: <http://purl.obolibrary.org/obo/BFO_0000050>
PREFIX biological_attribute: <http://purl.obolibrary.org/obo/OBA_0000001>
PREFIX morphology: <http://purl.obolibrary.org/obo/PATO_0000051>
PREFIX cardiovascular_system: <http://purl.obolibrary.org/obo/UBERON_0004535>
PREFIX tissue: <http://purl.obolibrary.org/obo/UBERON_0000479>

SELECT DISTINCT ?oba ?oba_label ?entity_label ?anatomy ?anatomy_label
WHERE  {
  ?oba subclass_of: biological_attribute: .
  ?oba subclass_of: morphology: .
  ?oba characteristic_of: ?entity .
  ?entity part_of: cardiovascular_system: .
  ?entity subclass_of: tissue: .

  ?entity label: ?entity_label .
  ?oba label: ?oba_label .
}
```
